# Computational Prediction of Blood-Brain Barrier Permeability Using Decision Tree Induction

**DOI:** 10.3390/molecules170910429

**Published:** 2012-08-31

**Authors:** Claudia Suenderhauf, Felix Hammann, Jörg Huwyler

**Affiliations:** 1 Division of Pharmaceutical Technology, Department of Pharmaceutical Sciences, University of Basel, Klingelbergstrasse 50, CH-4056 Basel, Switzerland; 2 Psychiatric Hospital of the University of Basel, Wilhelm-Klein-Str. 27, 4012 Basel, Switzerland

**Keywords:** blood brain barrier, drug transport, decision tree induction, QSAR modeling

## Abstract

Predicting blood-brain barrier (BBB) permeability is essential to drug development, as a molecule cannot exhibit pharmacological activity within the brain parenchyma without first transiting this barrier. Understanding the process of permeation, however, is complicated by a combination of both limited passive diffusion and active transport. Our aim here was to establish predictive models for BBB drug permeation that include both active and passive transport. A database of 153 compounds was compiled using *in vivo* surface permeability product (logPS) values in rats as a quantitative parameter for BBB permeability. The open source Chemical Development Kit (CDK) was used to calculate physico-chemical properties and descriptors. Predictive computational models were implemented by machine learning paradigms (decision tree induction) on both descriptor sets. Models with a corrected classification rate (CCR) of 90% were established. Mechanistic insight into BBB transport was provided by an Ant Colony Optimization (ACO)-based binary classifier analysis to identify the most predictive chemical substructures. Decision trees revealed descriptors of lipophilicity (aLogP) and charge (polar surface area), which were also previously described in models of passive diffusion. However, measures of molecular geometry and connectivity were found to be related to an active drug transport component.

## 1. Introduction

Experimental determination of blood-brain barrier (BBB) permeability for small molecules is notoriously difficult. In small experimental animals (*i.e*., mouse or rat), pharmacokinetic experiments are used to determine brain tissue clearance. The volume cleared per unit time is designated as the BBB permeability-surface area (PS) product (logPS), a parameter obtained from *in situ* brain perfusion studies in which a (radiolabeled) test compound is directly injected into the internal carotid artery [1,2,3]. This procedure is considered superior to other methods such as blood to brain drug partition measurements at steady state (logBB), as it lacks systemic distribution effects, which distort brain penetration substantially [4]. logPS is a complex parameter, because it encompasses passive transcellular diffusion across the BBB as well as a possible contribution by active transport. Small lipophilic agents (e.g., ethanol) cross the endothelial cell membrane by passive diffusion [5]. The process of passive permeation is well characterized [6,7,8,9]. According to Fick’s law of diffusion, the rate of passive diffusion of a small molecule across a phospholipid membrane will be proportional to the partition coefficient of the drug between the membrane and the external medium, the diffusion coefficient of the drug within the membrane and the concentration gradient across the membrane [10]. Major physico-chemical determinants for the process of membrane binding and diffusion are lipophilicity, molecular weight, and measures of molecular polarity [11]. However, such rules do not accurately reflect the complexity of membrane interactions *in vivo*, as they disregard non-specific membrane binding and biochemical processes mediated by transport proteins (*i.e*., facilitated transport or active transport) [12]. 

Typically, anticancer drugs, corticosteroids, and anti-epileptics are well-documented examples in which high passive cellular permeability is counteracted by an active drug efflux transport [13,14]. Physiologically, the involved ATP-binding cassette (ABC) transporters or solute carriers (SLC) mediate active transport across the BBB and constitute a biochemical barrier to protect brain tissue from potentially toxic compounds, such as blood borne xenobiotics. P-glycoprotein (MDR1 or P-gp) and breast cancer resistance protein (ABCG2 or BCRP) are the most prominent and best characterized representatives [15,16,17,18,19] and show the highest mRNA expression levels of all ABC-transporters of the human BBB [20]. Their impact on brain uptake of xenobiotics has been shown to be of clinical relevance [21]. Despite favorable molecular properties, central nervous system (CNS) concentrations of these drugs are significantly lower than expected. This results in suboptimal exposure and therefore poor pharmacological activity in the target tissue. 

As an alternative to invasive animal experiments, *in vitro* and *in silico* screening methods have been introduced to assist in the development of CNS active drugs. As compared to cell culture based assays, computational models provide a very high throughput and offer a mechanistic insight into molecular mechanisms of BBB transport. There are different strategies covering the use and application of such models. Calculated or measured physico-chemical properties may give first indications on the BBB permeability of a test compound. For example, compounds with a molecular weight less than 400–600 Da [11], a polar surface < 70 Å^2^ [22] and an octanol to water partition coefficient close to 3.4 [23] are said to have the potential to transit the BBB by passive diffusion. As opposed to such simplistic rules, more sophisticated *in silico* methods have be devised to establish statistical correlations between a given biological endpoint (such as blood-brain barrier permeability) and physico-chemical properties and molecular descriptors (for reviews see [24,25,26]). Here again, specific molecular properties can potentially be identified that favor BBB permeability.

In view of the shortcomings of existing computational models, the aim of the present project was as follows: first, a comprehensive and consistent data set of a complex but highly predictive biological endpoint (logPS) was compiled from literature data. In some instances, data from different literature sources were available. In case of differences, experimental protocols were analyzed and priority was given studies where standardized protocols were used. The final dataset comprised 153 compounds and is provided as Supplementary Information. Because the majority of data found in the literature was gathered in rats, we decided to omit data acquired in other species. We thus avoided interspecies variability and artifacts introduced by different surgical procedures as established, for example, in smaller animals such as mice. In particular, the ligation of the pterygoplatine artery, a small tributary, is done in the rat but is not possible in very small animals (*i.e*., mouse) [27,28,29].

Second, modern machine learning algorithms were applied to predict logPS values from calculated physico-chemical descriptors. We did not exclude drugs from our dataset that are suspected of being actively transported. This is in line with current practice, for example in the prediction of enzyme-drug interactions [26] or the discrimination between substrates, inhibitors, and inducers of P-glycoprotein [30]. Our model thus predicts brain penetration in general and thereby accounts for passive diffusion as well as a putative contribution by active transport. Third, computational tools and algorithms were selected with a focus on ease of use. Our dataset did not contain proprietary information. The final predictive model can be implemented easily, because it is based on open-source software packages that encourage free redistribution and access their design and implementation details. Fourth, the ant colony optimization (ACO) computing paradigm was used to identify relevant molecular substructures. Such motifs can be used to identify features of prototypic CNS drugs.

## 2. Results and Discussion

### 2.1. Data Set

The prerequisite for any QSAR modeling approach is the availability of a high quality dataset of biological endpoints. With respect to drug uptake into the central nervous system, comprehensive datasets have been established based on *in vivo* pharmacokinetic studies in which brain exposure is determined after intravenous peripheral administration of a test compound [9,31]. However, such blood to brain (logBB) drug partition measurements may be misleading due to drug metabolism and distribution in peripheral tissues [4]. In the present study, a dataset of 153 small molecules was therefore compiled (Supplementary Information, Appendix Table 1) using more reliable *in vivo* BBB permeability-surface area (logPS) products, which are obtained by direct internal carotid artery perfusion [32,33,34,35,36,37,38,39,40,41,42,43,44].

This method has the advantage of high sensitivity, as there is no systemic exposure of the test compound prior to its transport across the blood-brain barrier (BBB). Due to demanding and time consuming surgical and experimental procedures needed for this technology, our dataset can be considered high quality (but of comparably small size). In contrast to other studies, we focused on data from wild type animals and did not exclude suspected substrates of active transporters. Therefore, we were able to take into consideration a possible contribution by active transport. Active transport plays a major role in BBB permeation and can alter the pharmacokinetics of a drug substantially [45]. It is important to note that a contribution by active transport can be accounted for in our models, as demonstrated previously [30]. Moreover, one can hardly assure purity of a dataset if only passively transported molecules are included. The characterization of active transport mechanisms is still an ongoing topic of research and active transport mediated by yet unknown transporters may remain undetected when saturation occurs at very low concentrations. 

### 2.2. Chemical Space and Compound Classification

The low level of chemical similarity (Tanimoto coefficient = 0.282 for our dataset of n = 120 compounds used for classification learning) reflects the broad chemical space covered by our dataset. The range of physico-chemical properties of the dataset is indicated in Table 1.

**Table 1:** Range of physico-chemical properties of the dataset (n = 120) used for classification learning.

Parameter	Range of values
Molecular weight	46–1201 Da
Partition coefficient (aLogP)	−4.3–2.4
Polar surface area (tPSA)	3.2–279 Å^2^
Rotatable bonds count	0–18
Hydrogen bond acceptor count	1–23

In the past, criticism arose that binning into CNS positive and CNS negative substances is often based on presence or absence of pharmacological CNS activity, respectively [12]. We agree that pharmacological activity is a qualitative and inadequate measure of brain permeation ability, because the pharmacodynamic action of a compound is linked to unbound drug concentrations in the brain and not solely to its permeation ability. We therefore used a quantitative permeability measure (*i.e*., logPS) for classification. 

The paradigms used in the present study were classification algorithms. Data were therefore split in two classes, according to cut-off values published in literature [46,47]. The distinction of positively (CNSp+) and negatively (CNSp−) classified molecules refers to compounds with logPS values ≥ −2 and ≤−3, respectively. To achieve better separability and due to the scarcity of data points in this range, logPS values between −2.1 and −2.9 were exempt from classification learning. It should be noted that alternative splitting and classification schemes might be applied to the present dataset provided in Appendix, Table 1. In fact, inclusion of a third middle class with a reduced representativity (*i.e*., datapoints covering the range of logPS values between −2.1 and −2.9) will increase the complexity of the proposed models but might in return offer additional benefits.

### 2.3. Descriptors and Modeling

**Figure 1:**
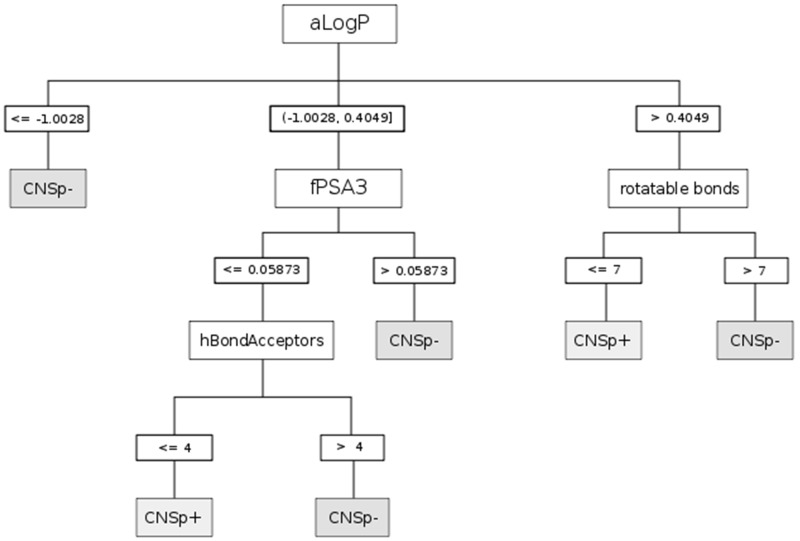
Decision tree built with the chi-squared automatic interaction detector (CHAID) on CDK descriptors. Prediction of strong (CNSp+, grey boxes) or weak (CNSp−, grey boxes) blood-brain barrier permeation is based on the splitting criteria (white boxes) of the partition coefficient (aLogP), rotatable bonds count, charge weighted partial positive surface area divided by total molecular surface area (fPSA3), and hydrogen bond acceptor count (hBondAcceptors).

Modern machine learning algorithms were applied to predict *in vivo* BBB permeability represented by logPS values. An initial survey of current machine learning methodologies, which have been applied to similar problems (e.g., regression analysis, support vector machines, artificial neural networks), yielded no well-performing models (data not shown) and lacked the interpretability of DTI and fragment-based analysis. The DTI paradigm is an efficient and powerful method to solve even linearly inseparable problems. Two widely used paradigms were used to induce decision trees. A first model (using the CHAID chi-squared automatic interaction detector) first described in 1964 [48], is shown in Figure 1.

The 10-fold cross-validated model achieved a high corrected classification rate (CCR) of 90.9% and a Matthews correlation coefficient (MCC) of 81.7%. Splitting criteria are summarized in Table 2. 

**Table 2:** Features revealed by decision tree induction (DTI) to predict brain penetration (logPS). Definition of selection criteria used for the DTI paradigms shown in Figure 1 and Figure 2.

Paradigm	Splitting criteria	Comment
CHAID	aLogP	Partition coefficient according to Ghoose-Crippen
	fPSA3	Charge weighted partial positive surface area/total molecular surface area
	hBondAcceptors	Hydrogen bond acceptor count
	rotatable bonds	Rotatable bonds count
CART	aLogP	Partition coefficient according to Ghoose-Crippen
	BCUTS	The number of highest eigenvalue, weighted for the lowest atom
	tPSA	Topological polar surface area

**Figure 2:**
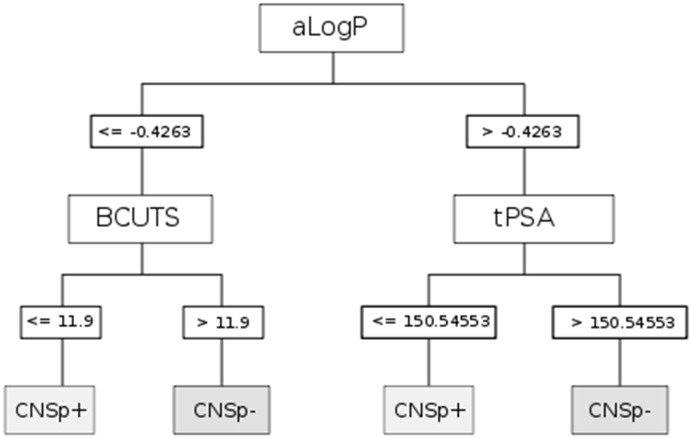
Classification and regression tree (CART) based on CDK descriptors. Prediction of strong (CNSp+, grey boxs) or weak (CNSp−, grey boxes) blood-brain barrier permeation is based on the splitting criteria (white boxes) of partition coefficient (aLogP), topological polar surface area (tPSA), and the number of highest eigenvalue of the Burden matrix weighted for the lowest atomic weight.

A second model, the CART classification and regression tree algorithm [49], achieved comparable performance (CCR: 89.8%, MCC: 79.9%). The corresponding tree and the decision criteria are shown in Figure 2 and Table 1. The number of instances can vary greatly among branches of a decision tree. In order to maintain the readability of the diagrams, information on the classification accuracy of individual nodes is not shown. However, overall model performance is provided by the composite measures of CCR and MCC. It should be noted that both applied performance measures, *i.e*., CCR and MCC, take into account falsely and correctly classified instances. This addresses the problem of performance being overestimated by methods that assign the majority class of the dataset to any new structure that they classify (*i.e*., overfitting bias). We are aware of the redundancy of these measures, but we decided to present both, for reasons of better comparability to other work.

Implementation of decision tree models is convenient and straightforward. Their output can be interpreted intuitively, which is an advantage when decision rules have to be implemented. Application of the models is illustrated in Table 3. The extracellular marker sucrose is a small and hydrophilic compound that is characterized by a high polar surface area. Based on the aLogP < −1.0028, the compound is immediately classified as CNSp− in the CHAID model. In contrast, the CNS active compound midazolam qualifies in the same model as CNSp+ due to its high lipophilicity and the low number of rotatable bonds. These results can be corroborated in the CART model, taking into consideration, for sucrose, its low aLogP value and a BCUTS > 11.9. Midazolam would again qualify as CNSp+ in CART based on a high aLogP value and a small topological polar surface area.

**Table 3:** Example of the implementation of decision tree models. Calculated physico-chemical descriptors are used for the prediction of strong (CNSp+) or weak (CNSp−) blood-brain barrier permeation of two test compounds.

Chemical structure	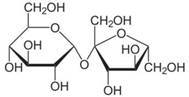	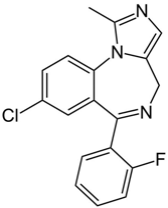
**Substance**	Sucrose	Midazolam
**Molecular weight**	342.12 Da	325.08 Da
**aLogP**	−4.3105	0.4073
**tPSA**	189.53 Å^2^	27.96 Å^2^
**fPSA3**	0.072136	0.033577
**Rotatable bonds**	5	1
**BCUTS**	11.9962	11.9974
**Prediction (CHAID/CART)**	CNSp−	CNSp+

It can be said that our models classified BBB permeability with excellent performance, but also provided profound insight into the biological processes involved. Interestingly, some of the features revealed by our models were also used in the past to predict passive brain permeation. Descriptors of lipophilicity and charge are frequently used to predict membrane permeation. It is therefore not surprising that three out of four paradigms selected the partition coefficient (aLogP) and/or polar surface area (fPSA3, tPSA) descriptors as splitting criteria. However, we found that both DTI paradigms set a much lower threshold for splitting on aLogP than earlier defined rules [6,50,51,52]. This could be an indicator of active transport involvement. Recent studies refer to increasing lipophilicity as a major rate-limiting feature for P-gp interactions and it played a predominant role in DTI models that predict P-gp inhibitors and substrates [30,53]. We were thus not able to confirm the assumption that high lipophilicity would be generally associated with good brain permeation [54]. While we found that it was clearly an important feature by which to split data, aLogP unfolded its predictive power for the present combined endpoint only in combination with other descriptors. 

Polar surface area was present in both models. Other groups observed a similar role of this feature in their work [54]. Generally, our models revealed that higher values for PSA corresponded with poor BBB permeation. The cutoff value for classification varied substantially between our models, but generally speaking, higher molecular polarity hindered passage into the hydrophobic milieu of the brain endothelial cells. The tree grown by CART used tPSA as a splitting criterion. Earlier work implies that PSA values over 60–90 Å^2^ are generally associated with poor brain permeation. In our model this threshold is much higher (150 Å^2^). Such a finding of a higher value was previously suspected to be a consequence of active transport [22,31,50]. Other groups reported an association between high polar interaction capacity and P-gp substrates [30,55]. Again, DTI paradigms seem to account for complex phenomena such as active transport. This is in agreement with previous findings and supports the applicability and validity of the present modeling approach [56,57].

CHAID predicted good BBB permeation for compounds with less than four hydrogen acceptors. This is an interesting finding, as it is generally agreed that hydrogen bond acceptors are less restrictive in terms of passive diffusion than are donors. Additionally, thresholds for classification were set by other authors at much higher levels (usually around 8 or 10) than our model suggests. One could argue that this finding could be an artefact emerging from the present dataset. However, we can see parallels to other work, where high hydrogen bond basicity was associated with P-gp substrates [58]. Accordingly, Norinder and Haeberlein reported that compounds exhibiting less than five nitrogen and oxygen entities would readily enter the brain [24]. This threshold corresponds with the cutoff value set in our model.

In the CHAID model, an increase in rotatable bonds was associated with poor BBB permeability. Interestingly, these findings contrast with work of Iyer *et al*. [54], who proposed an association between high molecular flexibility and increasing permeation ability. In addition, Iyer and colleagues refer to a proportional relationship of this feature with molecular weight. Consequently, an increase in rotatable bonds would infer a relationship between molecular weight and brain permeation. But in our dataset, we found a mediocre correlation between these two descriptors (R^2^ = 0.74). This finding is in accordance with the opinion of Abraham, who stated that molecular weight might not be as significant in predicting uptake into brain parenchyma as certain rules of thumb imply [59]. Diminished permeation ability with increasing number of rotatable bonds could also refer to potential conformational changes in molecular shape, *i.e*., an increase in bulkiness of a compound. Rotatable bonds are defined as any single bond not involved in a ring structure or connected to a non-terminal heavy atom. An extended conformation could roll up into a spherical and rather bulky shape. In other words, owing to its geometry, a molecule could potentially permeate the BBB to a lesser extent than its molecular weight would indicate. The number of rotatable bonds would then add additional information to models by also taking into account geometrical features rather than simply considering molecular mass. Similar correlations between molecular geometry (cross-sectional surface area) and BBB permeability have recently been reported [60]. The importance of geometry in predicting BBB penetration was substantiated by the use of BCUTS descriptors. Spectral indices such as mass weighted Burden matrix (BCUTS) refer to topology and complexity of a molecule as a whole.

### 2.4. Fragment Based Predictors

The question arises whether BBB permeating molecules in our dataset might share certain common molecular characteristics besides their numerical physic-chemical characteristics. We therefore performed a fragment-based analysis using an algorithm recently described by our group [61]. Ant colony optimization (ACO) is a natural computing paradigm introduced by Bonabeau *et al*. [62].The algorithm uses an abstraction of ant foraging behavior to find select, meaningful features. Higher-dimensional QSAR studies, e.g., ligand docking, routinely apply ACO alongside other optimization paradigms. With a few modifications, ACO can be used as a feature selector, *i.e*., to identify attributes that carry information about the endpoint of interest. In the present project, structural fingerprints were calculated and compared by binary ACO classification. The best performing subset of bits revealed by ACO are summarized in Table 4.

**Table 4:** Fingerprints selected from the MACCS keys are given along with their internal number (No), SMARTS code, and a short explanation of the substructure. In the sample structure, “A” stands for any atom, “X” for a heteroatom, and “R” for any molecular substructure.

No	Sample Structure	SMARTS	Description
23	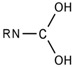	[#7]~[#6](~[#8])~[#8]	Nitrogen connected to carbon atom, which is connected to two oxygen atoms.
36	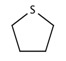	[#16R]	Any heterocycle containing a sulfur atom.
60	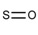	[#16]=[#8]	Oxygen and sulfur connected by a double bond.
82	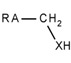	*~[CH2]~[!#6;!#1;!H0]	Any atom connected to CH_2_, which is itself connected to a heteroatom with at least one hydrogen atom.
122	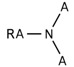	*~[#7](~*)~*	Any atom connected to nitrogen. Nitrogen has to be connected with any two additional atoms.
130	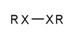	[!#6;!#1]~[!#6;!#1]	Two heteroatoms connected to each other.
145	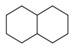	*1~*~*~*~*~*~1	Six ring structure, occurring twice in molecule. (They do not have to be directly connected)
150	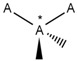	*!@*@*!@*	One intramolecular chirality center.
156	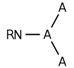	[#7]~*(~*)~*	Nitrogen connected to any three atoms.

This subset of chemical substructures achieved an acceptable CCR of 82.0% and a MCC of 0.64. The subset consisted of nine fingerprints selected from the MACCS key set (n = 166). 

**Figure 3:**
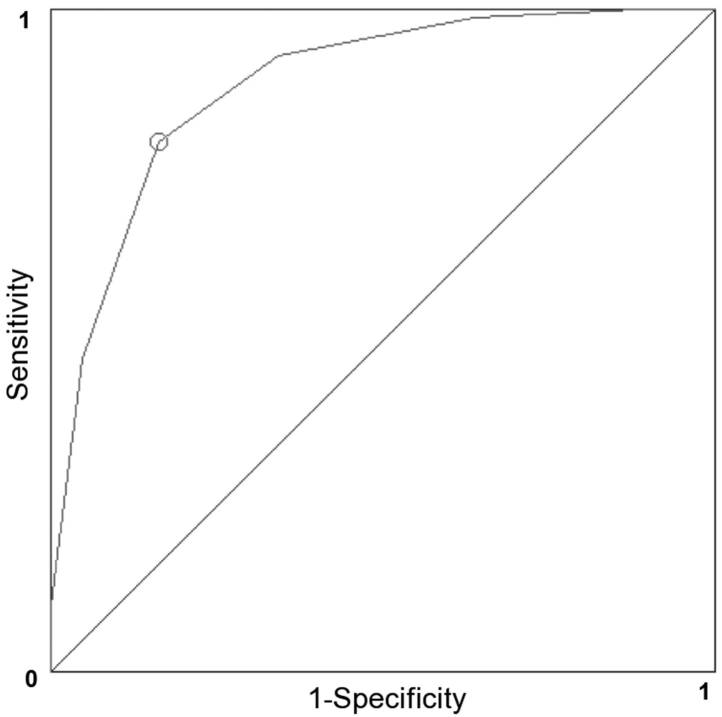
Analysis of molecular fingerprints according to receiver operating characteristic (ROC). Attributes were identified by ant colony optimization (ACO). Fingerprints were selected from the MACCS key set and consisted of nine fingerprints (Table 4). Cut-off value is denoted with a small circle.

Figure 3 shows the receiver operating characteristic (ROC) curve and cutoff point. The ROC analysis is a diagnostic tool by which true positive rate (sensitivity of the prediction) is plotted versus false positive rate (one minus the specificity or true negative rate). The corresponding area under the curve (AUC) was 0.89, indicating a high discrimination threshold of the binary classifier system. Selected fingerprints confirmed our findings from descriptor based machine learning (Table 4). The repeated inclusion of ring features indicates a strong contribution of lipophilicity, which is involved in passive and active transport processes across the BBB. Heteroatoms were present in seven out of nine fingerprints, of which four included explicitly nitrogen and/or oxygen atoms. This could relate, in analogy to our findings using DTI, to hydrogen bonding capacity and molecular polarity. However, an interesting structural feature was fingerprint No. 150, which refers to anticlockwise chirality. To our knowledge, stereoselectivity has not yet been used to predict BBB penetration ability. However, *in vivo* studies confirm involvement of stereoselectivity for drug transit across the BBB [63,64].

## 3. Experimental

### 3.1. Data Set

A dataset of 153 small molecules from literature was compiled containing information on *in vivo* BBB permeability-surface area (PS) products, usually given as logarithm (logPS) values determined in the rat species [32,33,34,35,36,37,38,39,40,41,42,43,44]. The complete dataset is provided as supplementary information (Appendix Table 1). There was no discrimination between passively transported molecules and suspected substrates of active transporters. logPS values ≥ −2 were judged as readily penetrating and received the label “CNSp+” (n = 65), while values ≤ −3 were labeled “CNSp−” (n = 55), or non-penetrating. Values between −2.1 and −2.9 were exempt from classification learning (n = 33). The final dataset consisted of 120 compounds. Structural information was retrieved from the National Library of Health database PubChem (http://pubchem.ncbi.nlm.nih.gov/). For salts, the counterion was removed. Stereochemical information are provided in all relevant locations. Conversion to three-dimensional structure representation was performed by using lowest energy conformers within the Ghemical force field [65].

### 3.2. Physicochemical Descriptors

Open source software was used to calculate 81 descriptors from the open source Chemical Development Kit (CDK; Version 1.2.3; http://cdk.sourceforge.net/) [66]. Chemical structures were encoded as SMILES (simplified molecular input line entry specification). Substructural patterns in molecules were specified using the SMARTS (SMiles ARbitrary Target Specification) notation. Three-dimensional structure-data representations required for conformational energy minimization were defined using Open Babel software (Version 2.3; http://openbabel.sourceforge.net/) [67].

### 3.3. Chemical Fingerprints

Chemical fingerprints are hash codes, evaluating the presence or absence of a list of substructural motifs (e.g. ketone groups, halogen atoms, *etc*.). The fingerprint darkness refers to the number of positive bits set, *i.e*., features found in the structure. The 166 bit MDL fingerprint key (MACCS) available in the Chemical Development Kit (CDK) was used [66].

### 3.4. Decision Tree Induction (DTI)

Decision trees were induced using two different paradigms. Chi-squared automatic interaction detector (CHAID) [48] selects attributes for splitting based on chi-squared testing. Classification and regression tree algorithm (CART) [49] uses the Gini coefficient [68] to find suitable splitting criteria. CHAID and CART were grown to a maximum depth of 3 and 5, respectively. We set minimum cases for parent nodes to 10 instances and allowed five cases in the child nodes. DTI was performed with PASW Statistics version 18 for Windows (http://www.spss.com/statistics/).

### 3.5. Ant Colony Optimization Classification (ACO)

A variant of ant colony optimization (ACO) classification, recently published by our group, was applied to gain specific structural insights [61]. The ACO paradigm selects fingerprint features of interests and tests their information gain with a heuristic fitness function. Quality measures of the fitness function are receiver operating characteristics (ROC), their areas under the curve (AUC), and additional parameters (CCR and MCC, see below). The Youdens J Index was used to determine the cut-off point in ROC curves [69].

### 3.6. Validation

A 10-fold cross-validation strategy was used to estimate performance of the models presented here [70]. The dataset was randomly divided into ten subsets, where nine sample folds were recombined for building the first tree which was tested against the remaining subset. This process is repeated ten times until all instances have been used for training and testing the model which is generally acknowledged to be a reasonable measure for model predictivity. Misclassification risk is estimated by applying the tree to the left out sample. The finally reported performance is calculated as the average of the risk of all trees generated. Although the here presented dataset spans a reasonable chemical space, it has to be pointed out that classification performance of compounds not covered in it could diverge from the here reported values as the final tree was not additionally validated with external data [71].

### 3.7. Chemical Similarity

Structural similarity of molecules is usually measured by assessing their distance in a multidimensional space spanned by their descriptors or fingerprints. MACCS fingerprints were compared using the Tanimoto coefficient available in the Open Babel tool kit [72]. This coefficient reports the average distance between all molecules. 

### 3.8. Quality Measures

As quality measures, the Corrected Classification Rate (CCR) and the Matthews Correlation Coefficient (MCC) were used as defined below: 


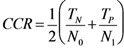


where T_N_ and T_P_ refer to compounds classified as true negative and true positive instances. N_0_ are all negative and N_1_ are all positive instances:


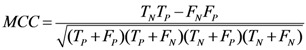


In the MCC formula, falsely negative (F_N_) and falsely positive (F_P_) classified molecules are additionally considered.

## 4. Conclusions

Decision tree induction is a convenient strategy to classify molecules according to their potential to permeate the blood-brain barrier. Due to their compactness, our DTI models are easily understood and have the potential to reconfirm our mechanistic understanding of the underlying processes involved in BBB permeation. Our models confirm the involvement of lipophilicity, size, and charge in predicting brain penetration. We were also able to identify additional contributing features such as molecular geometry, connectivity, stereochemistry, and relevant substructural motifs. Quite interestingly, our models seem to account for a potential involvement of active transport. One could argue that the data underlying our models were derived from rodents and might not accurately reflect the situation in humans. However, invasive BBB permeation measurements in man are not feasible. There is little data from intraoperative microdialysis experiments conducted in patients who underwent neurosurgery. But these reports most likely reflect pathophysiological conditions and are therefore inadequate to model the healthy blood-brain barrier. Therefore, the degree to which our models might reflect the situation in the human being and whether they might be adopted to predict BBB permeation in human remains to be elucidated. 

## Supplementary Materials

Supplementary materials can be accessed at: http://www.mdpi.com/1420-3049/17/9/10429/s1.
