# Supplementary material for: Computational Prediction of Blood-Brain Barrier Permeability Using Decision Tree Induction
Source: Molecules. 2012 Aug 31;17(9):10429–45. doi: 10.3390/molecules170910429 (PMC6269008; doi:10.3390/molecules170910429)
Supplement: Supplementary file 1 [file molecules-17-10429-s001.pdf]

Supplementary Material

Computational Prediction of Blood-Brain Barrier Permeability Using Decision Tree Induction

Claudia Suenderhauf [1], Felix Hammann [1,2] and Jörg Huwyler [1,*]

[1] Division of Pharmaceutical Technology, Department of Pharmaceutical Sciences, University of Basel, Klingelbergstrasse 50, CH-4056 Basel, Switzerland

[2] Psychiatric Hospital of the University of Basel, Wilhelm-Klein-Str. 27, 4012 Basel, Switzerland

* Author to whom correspondence should be addressed; E-Mail: joerg.huwyler@unibas.ch; Tel.: +41-61-267-15-00.

Appendix Table 1. Dataset (n = 153) of SMILES structural descriptors, blood-brain barrier LogPS values, classification and references.

Number	SMILES	LogPS	Classification	Reference [PMID]
1	C[C@H]1[C@H]([C@H](C[C@@H](O1)O[C@H]2C[C@@](CC3=C(C4=C(C(=C23)O)C(=O)C5=C(C4=O)C=CC=C5OC)O)(C(=O)CO)O)N)O	−5.7	CNSp−	18481317
2	C([C@@H]1[C@H]([C@@H]([C@H]([C@H](O1)O[C@]2([C@H]([C@@H]([C@H](O2)CO)O)O)CO)O)O)O)O	−5.3	CNSp−	9466345
3	C([C@H]([C@H]([C@@H]([C@@H](CO)O)O)O)O)O	−5.0	CNSp−	9466345
4	CC1=C2[C@H](C(=O)[C@@]3([C@H](C[C@@H]4[C@]([C@H]3[C@@H]([C@@](C2(C)C)(C[C@@H]1OC(=O)[C@@H]([C@H](C5=CC=CC=C5)NC(=O)C6=CC=CC=C6)O)O)OC(=O)C7=CC=CC=C7)(CO4)OC(=O)C)O)C)OC(=O)C	−4.7	CNSp−	18481317
5	COC1=CC(=CC(=C1OC)OC)[C@H]2[C@@H]3[C@H](COC3=O)[C@@H](C4=CC5=C(C=C24)OCO5)O	−4.6	CNSp−	18481317
6	CNS(=O)(=O)CC1=CC2=C(C=C1)NC=C2CCN(C)C	−4.6	CNSp−	17405866
7	C([C@H]([C@H](CO)O)O)O	−4.6	CNSp−	9466345
8	CN1CC(=O)N=C1N	−4.6	CNSp−	7392035
9	C1C[C@@H](O[C@@H]1CO)N2C=NC3=C2NC=NC3=O	−4.5	CNSp−	18481317
10	C1=CC(=C(C(=C1)O)N)C(=O)CC(C(=O)O)N	−4.5	CNSp−	19591936
11	CC(C)[C@@H](C(=O)O)N	−4.4	CNSp−	3468857

Appendix Table 1. *Cont.*

Number	SMILES	LogPS	Classification	Reference [PMID]
12	C[C@@H]1[C@H]([C@H](C[C@@H](O1)O[C@@H]2[C@H](O[C@H](C[C@@H]2O)O[C@@H]3[C@H](O[C@H](C[C@@H]3O)O[C@H]4CC[C@]5([C@@H](C4)CC[C@@H]6[C@@H]5C[C@H]([C@]7([C@@]6(CC[C@@H]7C8=CC(=O)OC8)O)C)O)C)C)C)O)O	−4.3	CNSp−	14709630
13	C[C@@]1(C(=O)N2[C@H](C(=O)N3CCC[C@H]3[C@@]2(O1)O)CC4=CC=CC=C4)NC(=O)[C@@H]5C[C@H]6[C@@H](CC7=CNC8=CC=CC6=C78)N(C5)C	−4.2	CNSp−	18481317
14	C1=CC=C(C(=C1)C(=O)C[C@@H](C(=O)O)N)N	−4.2	CNSp−	3819731
15	CC1=C(N=CN1)CSCCNC(=NC)NC#N	−4.1	CNSp−	10993764
16	C[C@H](CCC(=O)NCCS(=O)(=O)O)[C@H]1CC[C@@H]2[C@@]1([C@H](C[C@H]3[C@H]2[C@@H](C[C@H]4[C@@]3(CC[C@H](C4)O)C)O)O)C	−4.1	CNSp−	14709630
17	CC(C)C[C@@H](C(=O)O)N	−4.1	CNSp−	3468857
18	C1CCC(CC1)(C(=O)O)N	−4.0	CNSp−	18481317
19	CC1=CN(C(=O)NC1=O)[C@H]2C[C@@H]([C@H](O2)CO)N=[N+]=[N-]	−4.0	CNSp−	9593963
20	C[N+](C)(C)CCO	−4.0	CNSp−	18481317
21	C1[C@H]2[C@@H]([C@@H](S1)CCCCC(=O)O)NC(=O)N2	−4.0	CNSp−	18481317
22	C1=NC2=C(N1)C(=S)N=CN2	−4.0	CNSp−	18481317
23	CN(CC1=CN=C2C(=N1)C(=NC(=N2)N)N)C3=CC=C(C=C3)C(=O)N[C@@H](CCC(=O)O)C(=O)O	−3.9	CNSp−	14709630
24	CCCN(CCC)S(=O)(=O)C1=CC=C(C=C1)C(=O)O	−3.9	CNSp−	18481317
25	CC1=CN(C(=O)NC1=O)[C@H]2C[C@@H]([C@H](O2)CO)O	−3.8	CNSp−	9593963
26	C1=NC2=C(N1)C(=O)NC(=O)N2	−3.8	CNSp−	14709630
27	C(C(=O)O)I	−3.8	CNSp−	18481317
28	CC1=C(C(=C(C2=C1N(C=C(C2=O)C(=O)O)C3CC3)N)F)N4C[C@H](C5(C4)CC5)N	−3.8	CNSp−	18481317
29	C(CC(=O)N)C(C(=O)O)N	−3.8	CNSp−	18481317
30	C(=O)(N)N	−3.8	CNSp−	9466345
31	CN(C)CCC1=CNC2=C1C=C(C=C2)CN3C=NC=N3	−3.8	CNSp−	17405866

Appendix Table 1. *Cont.*

32	CC1=C2[C@H](C(=O)[C@@]3([C@H](C[C@@H]4[C@]([C@H]3[C@@H]([C@@](C2(C)C)(C[C@@H]1OC(=O)[C@@H]([C@H](C5=CC=CC=C5)NC(=O)C6=CC=CC=C6)O)O)OC(=O)C7=CC=CC=C7)(CO4)OC(=O)C)O)C)OC(=O)CCC(=O)O	−3.7	CNSp−	18481317
33	CC1CN(CCN1)C2=C(C(=C3C(=C2)N(C=C(C3=O)C(=O)O)C4CC4)C)F	−3.7	CNSp−	10991972
34	CC1([C@H](C(=O)NCC(=O)N[C@H](C(=O)N[C@H](C(SS1)(C)C)C(=O)O)CC2=CC=CC=C2)NC(=O)[C@H](CC3=CC=C(C=C3)O)N)C	−3.6	CNSp−	14709630
35	C(=N)(N)N	−3.6	CNSp−	18481317
36	CC[C@@]1(C[C@@H]2C[C@@](C3=C(CCN(C2)C1)C4=CC=CC=C4N3)(C5=C(C=C6C(=C5)[C@]78CCN9[C@H]7[C@@](C=CC9)([C@H]([C@@]([C@@H]8N6C)(C(=O)OC)O)OC(=O)C)CC)OC)C(=O)OC)O	−3.5	CNSp−	10993764
37	C1=CC(=C(C=C1C2=C(C(=O)C3=C(C=C(C=C3O2)O)O)O)O)O	−3.5	CNSp−	14980703
38	C(C(=O)O)N	−3.5	CNSp−	14709630
39	C1=NC2=C(N1)C(=O)N=CN2	−3.5	CNSp−	14709630
40	C(C(=O)O)C(=O)O	−3.5	CNSp−	18481317
41	C1=CC=C(C=C1)C2C(=O)N=C(O2)N	−3.5	CNSp−	17405866
42	CC(C(=O)O)N	−3.4	CNSp−	10993764
43	COC1=CC2=C(C=CN=C2C=C1)[C@@H]([C@H]3CC4CCN3C[C@@H]4C=C)O	−3.4	CNSp−	14709630
44	C1=CC=C(C(=C1)C(=O)O)O	−3.4	CNSp−	14709630
45	C(=S)(N)N	−3.4	CNSp−	9466345
46	CC(=O)N[C@H]1CCC2=CC(=C(C(=C2C3=CC=C(C(=O)C=C13)OC)OC)OC)OC	−3.3	CNSp−	14709630
47	C1=CC(=CC=C1C[C@@H](C(=O)O)N)N(CCCl)CCCl	−3.3	CNSp−	3815357
48	C(CS(=O)(=O)O)N	−3.2	CNSp−	18481317
49	C1CCC(CC1)(CC(=O)O)CN	−3.2	CNSp−	18481317
50	CC[C@@]1(C[C@@H]2C[C@@](C3=C(CCN(C2)C1)C4=CC=CC=C4N3)(C5=C(C=C6C(=C5)[C@]78CCN9[C@H]7[C@@](C=CC9)([C@H]([C@@]([C@@H]8N6C=O)(C(=O)OC)O)OC(=O)C)CC)OC)C(=O)OC)O	−3.1	CNSp−	18481317

Appendix Table 1. *Cont.*

51	C1CC(OC1)N2C=C(C(=O)NC2=O)F	−3.1	CNSp−	18481317
52	CCCC(C)(COC(=O)N)COC(=O)N	−3.1	CNSp−	17405866
53	CN1C=NC2=C1C(=O)NC(=O)N2C	−3.0	CNSp−	14709630
54	C1C2CC3CC1CC(C2)(C3)N	−3.0	CNSp−	18481317
55	CC1=C(SC=[N+]1CC2=CN=C(N=C2N)C)CCO	−3.0	CNSp−	18481317
56	C(CO)O	−2.9	exempt	9466345
57	COC1=CC=CC2=C1NC3=C(C2=O)CCCCC3	−2.9	exempt	18481317
58	COCC(CN1C=CN=C1[N+](=O)[O-])O	−2.9	exempt	18481317
59	CN(CC[C@H](C1=CC=C(C=C1)F)OC2=CC=C(C=C2)C3=CC=CC=C3)CC(=O)O	−2.9	exempt	14709630
60	CN1C2=C(C(=O)N(C1=O)C)NC=N2	−2.9	exempt	14709630
61	CCCCC[C@@H](/C=C/[C@H]1[C@@H](C[C@@H]([C@@H]1C/C=C\CCCC(=O)O)O)O)O	−2.9	exempt	18481317
62	CCCCNC(=O)NS(=O)(=O)C1=CC=C(C=C1)C	−2.8	exempt	18481317
63	C1CC(=O)NC(=O)C1NC(=O)N(CCCl)N=O	−2.8	exempt	18481317
64	COC1=C(C=C(C=C1)Cl)C(=O)NCCC2=CC=C(C=C2)S(=O)(=O)NC(=O)NC3CCCCC3	−2.8	exempt	10993764
65	CO[C@H]1[C@@H]([C@H](OC([C@@H]1O)O)CO)O	−2.8	exempt	18481317
66	C[C@@H]1[C@H]2CC3=C([C@@]1(CCN2CC=C(C)C)C)C=C(C=C3)O	−2.8	exempt	18481317
67	CC1=NC=C(N1CCO)[N+](=O)[O-]	−2.7	exempt	18481317
68	C[C@H]1[C@H]([C@H](C[C@@H](O1)O[C@H]2C[C@@](CC3=C(C4=C(C(=C23)O)C(=O)C5=C(C4=O)C=CC=C5OC)O)(C(=O)C)O)N)O	−2.7	exempt	14709630
69	CN1CC[C@]23[C@@H]4[C@H]1CC5=C2C(=C(C=C5)O)O[C@H]3[C@H](C=C4)O	−2.7	exempt	14709630
70	C1=CC(=C(C(=C1)Cl)Cl)C2=C(N=C(N=N2)N)N	−2.7	exempt	17405866
71	CCN(CC)CCNC(=O)C1=CC(=C(C=C1OC)N)Cl	−2.7	exempt	17405866
72	C1=CC=C(C=C1)CCNN	−2.7	exempt	17405866
73	C1C(OC2=CC(=CC(=C2C1=O)O)O)C3=CC=C(C=C3)O	−2.6	exempt	14980703
74	CCCC(CCC)C(=O)O	−2.5	exempt	14709630
75	CN1C=C(C2=CC=CC=C21)C(=O)[C@@H]3CCC4=C(C3)NC=N4	−2.5	exempt	18481317

Appendix Table 1. *Cont.*

76	C(C[C@@H](C(=O)O)N)CN=C(N)N	−2.5	exempt	18481317
77	CCC1(CC(=O)NC1=O)C	−2.5	exempt	17405866
78	CC1=C(SC=C1)C(=CCCN2CCC[C@H](C2)C(=O)O)C3=C(C=CS3)C	−2.5	exempt	17405866
79	C1=NC2=C(C(=N1)N)N=CN2[C@H]3[C@@H]([C@@H]([C@H](O3)CO)O)O	−2.4	exempt	9593963
80	CSCC[C@@H](C(=O)O)N	−2.4	exempt	18481317
81	CCN(C1=CC=CC(=C1)C2=CC=NC3=C(C=NN23)C#N)C(=O)C	−2.3	exempt	17405866
82	C([C@@H]1[C@H]([C@@H]([C@H](C(O1)O)O)O)O)O	−2.2	exempt	10993764
83	C1=CC=C(C=C1)C2(C(=O)NC(=O)N2)C3=CC=CC=C3	−2.2	exempt	14709630
84	CC1=C(C2=C(N1C(=O)C3=CC=C(C=C3)Cl)C=CC(=C2)OC)CC(=O)O	−2.2	exempt	18481317
85	CC(CC1=CC=CC=C1)(C2=CC=CC=C2)NC(=O)CN	−2.2	exempt	18481317
86	C1=CC(=C(C=C1CCN)O)O	−2.1	exempt	14709630
87	C[C@@]1(C(=O)N2[C@H](C(=O)N3CCC[C@H]3[C@@]2(O1)O)CC4=CC=CC=C4)NC(=O)[C@H]5CN([C@@H]6CC7=CNC8=CC=CC(=C78)C6=C5)C	−2.1	exempt	17405866
88	C1CCC2=NC3=CC=CC=C3C(=C2C1)N	−2.1	exempt	17405866
89	C1=CC=C(C=C1)NC(=S)N	−2.0	CNSp+	18481317
90	CC(=O)CC(C1=CC=CC=C1)C2=C(OC3=CC=CC=C3C2=O)O	−2.0	CNSp+	10993764
91	CC1=CC(=O)N(N1C)C2=CC=CC=C2	−2.0	CNSp+	14709630
92	CN1C=NC2=C1C(=O)N(C(=O)N2C)C	−2.0	CNSp+	14709630
93	CN(C)CCCC1(C2=C(CO1)C=C(C=C2)C#N)C3=CC=C(C=C3)F	−2.0	CNSp+	17405866
94	CN(C)CC(C1=CC=C(C=C1)OC)C2(CCCCC2)O	−2.0	CNSp+	17405866
95	CC1=CNC(=O)NC1=O	−1.9	CNSp+	9466345
96	CC[C@H]1C(=O)N(CC(=O)N([C@H](C(=O)N[C@H](C(=O)N([C@H](C(=O)N[C@H](C(=O)N[C@@H](C(=O)N([C@H](C(=O)N([C@H](C(=O)N([C@H](C(=O)N([C@H](C(=O)N1)[C@@H]([C@H](C)C/C=C/C)O)C)C(C)C)C)CC(C)C)C)CC(C)C)C)C)C)CC(C)C)C)C(C)C)CC(C)C)C)C	−1.9	CNSp+	10993764
97	CCCCC/C=C\C/C=C\CCCCCCCC(=O)O	−1.9	CNSp+	18481317
98	CCN(CC)CC(=O)NC1=C(C=CC=C1C)C	−1.9	CNSp+	2338660
99	CN1CCN(CC1)CCCN2C3=CC=CC=C3SC4=C2C=C(C=C4)C(F)(F)F	−1.9	CNSp+	17405866

Appendix Table 1. *Cont.*

100	C1CN(CCN1CCCN2C3=CC=CC=C3SC4=C2C=C(C=C4)C(F)(F)F)CCO	−1.9	CNSp+	17405866
101	CN1CCCCC1CCN2C3=CC=CC=C3SC4=C2C=C(C=C4)S(=O)C	−1.8	CNSp+	17405866
102	CC1=C(C(=O)N2CCCCC2=N1)CCN3CCC(CC3)C4=NOC5=C4C=CC(=C5)F	−1.8	CNSp+	17405866
103	CCC1=C(C(=NC(=N1)N)N)C2=CC(=C(C=C2)Cl)Cl	−1.8	CNSp+	18481317
104	C1=CC(=C(C=C1C[C@@H](C(=O)O)N)O)O	−1.8	CNSp+	14709630
105	CCCCCCCC(=O)O	−1.8	CNSp+	18481317
106	CC(C1=CC(=C(C=C1)C2=CC=CC=C2)F)C(=O)O	−1.8	CNSp+	16715377
107	C1=CC=C2C(=C1)C=CC3=CC=CC=C3N2C(=O)N	−1.8	CNSp+	17405866
108	C1CN(CCN1CCC2=C(C=C3C(=C2)CC(=O)N3)Cl)C4=NSC5=CC=CC=C54	−1.7	CNSp+	17405866
109	CNC(=S)NC1=CC=CC=C1	−1.7	CNSp+	18481317
110	C1CC2([C@H]3CC4=C5[C@@]2(CCN3CC6CC6)[C@@H]([C@@H]1F)OC5=C(C=C4)O)O	−1.7	CNSp+	18481317
111	COC1=C(C=C2C(=C1O)C(=O)C=C(O2)C3=CC=C(C=C3)O)O	−1.7	CNSp+	18481317
112	CC(C)O	−1.7	CNSp+	9466345
113	CC1=C(N(N=C1C(=O)NN2CCCCC2)C3=C(C=C(C=C3)Cl)Cl)C4=CC=C(C=C4)Cl	−1.6	CNSp+	14709630
114	C1=CC(=CC=C1CCCC(=O)O)N(CCCl)CCCl	−1.6	CNSp+	14709630
115	CC(CC1=CC=CC=C1)(C2=CC=CC=C2)N	−1.6	CNSp+	18481317
116	CC(C)CC1=CC=C(C=C1)C(C)C(=O)O	−1.6	CNSp+	16715377
117	CCO	−1.5	CNSp+	9466345
118	CC(C(=O)C1=CC(=CC=C1)Cl)NC(C)(C)C	−1.5	CNSp+	17405866
119	CNCCC(C1=CC=CC=C1)OC2=CC=C(C=C2)C(F)(F)F	−1.5	CNSp+	14709630
120	COC1=C(C=C2C(=C1)CC(C2=O)CC3CCN(CC3)CC4=CC=CC=C4)OC	−1.5	CNSp+	17405866
121	C1CN(CCN1CCCN2C(=O)N3C=CC=CC3=N2)C4=CC(=CC=C4)Cl	−1.5	CNSp+	17405866
122	C1CN(CCC1(C2=CC=C(C=C2)Cl)O)CCCC(=O)C3=CC=C(C=C3)F	−1.5	CNSp+	17405866
123	CN1CCN(CC1)C2=NC3=CC=CC=C3OC4=C2C=C(C=C4)Cl	−1.5	CNSp+	17405866

Appendix Table 1. *Cont.*

124	CC1=CC=CC=C1O[C@H](CCNC)C2=C C=CC=C2	−1.4	CNSp+	17405866
125	CN1CCN2C(C1)C3=CC=CC=C3CC4=C 2N=CC=C4	−1.4	CNSp+	17405866
126	C[C@H](CC1=CC=CC=C1)N(C)CC#C	−1.4	CNSp+	17405866
127	CN1C2=C(C=C(C=C2)Cl)C(=NC(C1=O) O)C3=CC=CC=C3	−1.4	CNSp+	17405866
128	CNCCCC12CCC(C3=CC=CC=C31)C4= CC=CC=C24	−1.4	CNSp+	17405866
129	CN1CCCCC1CCN2C3=CC=CC=C3SC4 =C2C=C(C=C4)SC	−1.4	CNSp+	17405866
130	CN1C(=O)CN=C(C2=C1C=CC(=C2)Cl) C3=CC=CC=C3	−1.4	CNSp+	17405866
131	C1CN(CCN1CCOCCO)C2=NC3=CC=C C=C3SC4=CC=CC=C42	−1.3	CNSp+	17405866
132	CN(C)CC/C=C/1\C2=CC=CC=C2COC3 =CC=CC=C31	−1.3	CNSp+	17405866
133	C1=CC=C(C=C1)CC(C(=O)O)N	−1.3	CNSp+	18481317
134	CC[C@H](C)C(=O)O[C@H]1C[C@H](C=C2[C@H]1[C@H]([C@H](C=C2)C)C C[C@@H]3C[C@H](CC(=O)O3)O)C	−1.3	CNSp+	18481317
135	CC1=CC2=C(NC3=CC=CC=C3N=C2S1) N4CCN(CC4)C	−1.3	CNSp+	17405866
136	CN1CCN(CC1)C2=C3C=CC=CC3=NC4 =C(N2)C=C(C=C4)Cl	−1.3	CNSp+	17405866
137	C1CN(CCN1CCCN2C3=CC=CC=C3SC4 =C2C=C(C=C4)Cl)CCO	−1.3	CNSp+	17405866
138	CC1=CC(=NO1)C(=O)NNCC2=CC=CC =C2	−1.2	CNSp+	17405866
139	CC(C)NCC(COC1=CC=CC2=CC=CC=C 21)O	−1.2	CNSp+	14709630
140	CN(C)CCCN1C2=CC=CC=C2SC3=C1C =C(C=C3)Cl	−1.2	CNSp+	17405866
141	C1CN(CCN1)C2=NC3=CC=CC=C3OC4 =C2C=C(C=C4)Cl	−1.2	CNSp+	17405866
142	CN1CCN(CC1)CC/C=C/2\C3=CC=CC= C3SC4=C2C=C(C=C4)S(=O)(=O)N(C)C	−1.2	CNSp+	17405866
143	CC1=C(C(=O)N(N1C)C2=CC=CC=C2)I	−1.1	CNSp+	10993764
144	C[C@]12CC[C@H]3[C@H]([C@@H]1 CC[C@@H]2O)CCC4=CC(=O)CC[C@] 34C	−1.1	CNSp+	14709630
145	CN(C)CCC=C1C2=CC=CC=C2CCC3=C C=CC=C31	−1.1	CNSp+	17405866
146	CC1=NC=C2N1C3=C(C=C(C=C3)Cl)C(=NC2)C4=CC=CC=C4F	−1.0	CNSp+	18481317

Appendix Table 1. *Cont.*

147	C1=CC=C(C=C1)C2=NC(C(=O)NC3=C2C=C(C=C3)Cl)O	−1.0	CNSp+	18481317
148	C1C(=O)NC2=C(C=C(C=C2)Cl)C(=N1)C3=CC=CC=C3	−1.0	CNSp+	18481317
149	CCC(C)(C)C(=O)O[C@H]1C[C@H](C=C2[C@H]1[C@H]([C@H](C=C2)C)CC[C@@H]3C[C@H](CC(=O)O3)O)C	−1.0	CNSp+	18481317
150	CCCNC(=S)NC1=CC=CC=C1	−1.0	CNSp+	18481317
151	CCCN1C[C@@H](C[C@H]2[C@H]1CC3=CNC4=CC=CC2=C34)CSC	−1.0	CNSp+	17405866
152	C[C@]12CC[C@H]3[C@H]([C@@H]1CC[C@@H]2O)CCC4=C3C=CC(=C4)O	−0.8	CNSp+	9466345
153	CN[C@H]1CC[C@H](C2=CC=CC=C12)C3=CC(=C(C=C3)Cl)Cl	−0.7	CNSp+	17405866
